# Epigenetic clocks and female fertility timeline: A new approach to an old issue?

**DOI:** 10.3389/fcell.2023.1121231

**Published:** 2023-03-21

**Authors:** Letizia Li Piani, Paola Vigano', Edgardo Somigliana

**Affiliations:** ^1^ Department of Clinical Sciences and Community Health, Università Degli Studi di Milano, Milan, Italy; ^2^ Infertility Unit, Fondazione IRCCS Ca’ Granda Ospedale Maggiore Policlinico, Milan, Italy

**Keywords:** epigenetic, aging, infertility, pregnancy, epigenetic clock, newborn

## Abstract

Worldwide increase in life expectancy has boosted research on aging. Overcoming the concept of chronological age, higher attention has been addressed to biological age, which reflects a person’s real health state, and which may be the resulting combination of both intrinsic and environmental factors. As epigenetics may exert a pivotal role in the biological aging, epigenetic clocks were developed. They are based on mathematical models aimed at identifying DNA methylation patterns that can define the biological age and that can be adopted for different clinical scopes (i.e., estimation of the risks of developing age-related disorders or predicting lifespan). Recently, epigenetic clocks have gained a peculiar attention in the fertility research field, in particular in the female counterpart. The insight into the possible relations between epigenetic aging and women’s infertility might glean additional information about certain conditions that are still not completely understood. Moreover, they could disclose significant implications for health promotion programs in infertile women. Of relevance here is that the impact of biological age and epigenetics may not be limited to fertility status but could translate into pregnancy issues. Indeed, epigenetic alterations of the mother may transfer into the offspring, and pregnancy itself as well as related complications could contribute to epigenetic modifications in both the mother and newborn. However, even if the growing interest has culminated in the conspicuous production of studies on these topics, a global overview and the availability of validated instruments for diagnosis is still missing. The present narrative review aims to explore the possible bonds between epigenetic aging and fertility timeline. In the “infertility” section, we will discuss the advances on epigenetic clocks focusing on the different tissues examined (endometrium, peripheral blood, ovaries). In the “pregnancy” section, we will discuss the results obtained from placenta, umbilical cord and peripheral blood. The possible role of epigenetic aging on infertility mechanisms and pregnancy outcomes represents a question that may configure epigenetic clock as a bond between two apparently opposite worlds: infertility and pregnancy.

## 1 Introduction

The female reproductive timeline represents a shadowy and attractive topic of research. Albeit with individual variations and peculiarities, four milestones can be identified in the female reproductive lifespan: 1) menarche, corresponding to the beginning of fertile phase; 2) gestation, the fulfilment of the potential fertility; 3) the end of the fertile period occurring at about 10 years prior to menopause, and 4) menopause itself, marking the end of the ovarian hormonal activity and the onset of the senior age (te Velde and Pearson, 2002). The improved life expectancy and the social trend of postponing pregnancy have determined an increase in the average age of pregnant women, but also an increase in the rate of age-related infertility, boosting the interest on this topic (Mills et al., 2011; ESHRE Capri workshop group, 2017).

In the aging research field, the concept of biological age has recently gained major attention since it may better reflect the real physiological status and function of an individual compared to chronological age (Lowsky et al., 2014). Epigenetics, defined as genome modifications that do not involve changes in DNA sequence but rather modifications of chromatin, is a crucial determinant of biological age (Bell et al., 2019; López-Otín et al., 2013). The growing awareness of the role of epigenetics in aging has encouraged the introduction of several estimators of biological age. Among them, epigenetic clocks have been heralded as the most accurate. It is possible that these clocks may glean additional information regarding fertility timeline, in which aging exerts a pivotal part. The possible impact of epigenetic aging on infertility mechanisms, but also the epigenetic modifications during gestation and the impact of pregnancy on a woman’s biological age are intriguing queries. Within an evolutionary framework, aging and reproduction are indeed intrinsically linked (Levine et al., 2018).

The present narrative review considers the interaction between epigenetic clocks and fertility timeline. We mainly aimed to provide an overview of the knowledge gained on the role of epigenetic clocks in two apparent opposite faces of fertility, that is infertility and pregnancy. Table 1 summarizes the main features of the studies discussed in the present review. 

**TABLE 1:** Main characteristics of considered studies.

Infertility							
Author, year	Country	Study design	Target population	Tissue	Epigenetic clock	Age (ys)	Results
Olesen et al. (2018)	Denmark	Cross-sectional	9 volunteers from hospital	Endometrium	Horvath clock 2013	19.1–38.6	Endometrium was 1.5 ys older than blood (*p* = 0.23) and 4.4 ys than chronological age (*p* = 0.012)
Kovalenko et al. (2021)	Russia	Cross-sectional	236 patients from hospital	Endometrium	PTENP1 methylation rate	49	PTENP1 methylation rate increase to prevent malignant transformation of endometrial tissue and with approaching menopause
Monseur et al. (2020)	United States of America	prospective cohort	39 infertile women	Peripheral blood	Horvath clock 2013	38.0	Positive AgeAccel had lower AMH (p0.053), lower oocyte yield (p 0.0020) lower AFC (p 0.050)
Christensen et al. (2021)	Denmark	Case-control	55 women EOA vs. 52 NOA	Peripheral blood	Skin-Blood clock PhenoAge clock	32.8 vs. 29.3	EOA were 0.55 ys older than NOA (p 0.27). Similar results in PhenoAge and Skin-Blood clock
Li Piani et al. (2022)	Italy	Prospective cohort	181 infertile women	Peripheral blood	Zbieć-Piekarska clock	37.9	Women with LB were epigenetically younger than without LB (36.1 ± 4.2 vs. 37.3 ± 3.3 years, p 0.04)
Lee et al. (2022)	Norway	Case-control	1,000 spontaneous vs. 894 ART pregnant women	Peripheral blood	Horvath clock 2013 PhenoAge Hannum clock DunedinPoAm	30.1 vs. 33.57	DunedinPoAm acceleration was higher in IVF vs. non-ART mothers (0.021 years, *p*-value <0.0001) No significant results with the other clocks
Morin et al. (2018)	United States of America	Prospective cohort	A. 20 young good, B. 20 young poor, C. 20 older physiologic poor, D. 17 older unexpected good responders	Peripheral blood Cumulus cells	Horvath clock 2013	32.3 33.2 41.9 41.8	Epigenetic age of CC was younger than WBC samples from the same patient (*p* < 0.001). The predicted age in CCs was 9.3 years old on average, in respect of the group
Hanson et al. (2020)	United States of America	Prospectivec cohort	38 poor responders vs. 107 good responders	Peripheral blood Cumulus cells	Horvath clock 2013	37.7 vs. 34.6	The predicted age in CCs was 8.6 ys old on average in respect of the group
Olsen et al. (2018)	Denmark	multicenter cohort study	118 infertile women 63 MGC	Peripheral blood MGC	Horvath clock 2013 Skin&Blood Granulosa Clock	33.9	MGC age was 2.7 ys or 6.8 ys on average (Skin&Blood, Horvath clock) Good performance of the new granulosa clock
Olsen et al. (2018)	Denmark	multicenter cohort study	28 poor responders vs. 63 normoresponders vs. 28 high responders	Peripheral blood MGC	Skin&Blood Granulosa Clock	35.5 vs. 33.2 vs. 33.8	For both clocks and tissue types, no association between age acceleration and ovarian reserve. Epimutations higher in MGC of poor responders
Knight et al. (2022)	United States of America	Cross-sectional	70 infertile women	MGC	Horvath clock 2013 Granulosa Clock PhenoAge GrimAge	36.7	GrimAge acceleration was negatively associated with AMH levels (p 0.003) and AFC (p 0.0001)

Pregnancy							
Author, year	Country	Study design	Target population	Tissue	Epigenetic clock	Age (ys)	Results
Mayne et al. (2017)	Australia	Observational study	387 placentas from 12 datasets	placenta	Placenta GA clock from uncomplicated pregnancies. 62 CpGs identified	-	PAA was higher in case of early onset preeclampsia cases
Lee et al. (2019)	Norway	Observational study	1,102 placental samples	placenta	Placenta GA clock from complicated pregnancies. 558 CpGs identified	-	Robust, well-suited clock for GA estimation, even in pregnancy complications
Tekola-Ayele et al. (2019)	United States of America	Observational study	312 placenta samples	Placenta	Placenta GA clock	-	No significant sex differences in gestational age. PAA decreased with male fetal weight and increased with female fetal weight. A 1-week increase in PAA was associated with a double increase for low birth weight in males
Bohlin et al. (2016)	Norway	Cross-sectional	1753 newborns	Cord blood	Cord blood GA clock based on 223 CpG sites. Trained on 108 samples	-	GA clock results accurate in GA prediction according to CRL.
Girchenko et al. (2017)	Finland	Cross-sectional	814 mother-child	Cord blood	Cord blood Knight GA clock	-	Newborn GAA was associated with adverse maternal risk factors
Haftorn et al. (2021)	Norway	Case-control study	200 vs. 838 non ART vs. ART newborns	Cord blood	EPIC clock, based on 176 CpGs sites	-	EPIC clock was more accurate in GA prediction (*R* ^2^ = 0.724) than Bojlin and Knight ones. Same result in ART and non-ART newborns
Knight et al. (2016)	United States of America	Cross-sectional	1,434 newborns	Cord blood	Cord blood GA clock based on 148 CpG sites. Trained on 16,676 CpGs	-	GA acceleration significantly predicted birthweight (*p* = 0.033) after correcting for clinically estimated GA, race, cell type proportions, and cohort
Ross et al. (2020)	Canada	Longitudinal cohort	77 pregnant women	Peripheral blood	GrimAge	33.1	Higher maternal prenatal GrimAgeAccel with lower birthweight (p 0.001)
Madden et al. (2021)	Scotland	Cross-sectional	1757 adults	Peripheral blood	EWAS; Horvath; PhenoAge; Hannum; GrimAge; DNAmTL	37.1	EWAS study reveals higher birthweight along with higher methylation levels at CASZ1. Only DNAmTL and GrimAge correlates with birthweight
Mathewson et al. (2021)	Canada	Longitudinal case-control	45 ELBW vs. 47 NBW adult survivors	buccal epithelial cells	Horvath clock 2013	32.35–32.44	ELBW survivors were characterized by an increased rate of aging according to Horvath clock than NBW controls. For each additional adult risk, ELBW survivors were 2.16 ys epigenetically older than controls
Falick Michaeli et al. (2019)	Israel	Prospective	41 cord blood samples	Cord blood	New epigenetic clock based on RRBS	-	High correlation with GA (r = 0.77, *p*-value = 1.48E-05). Advantagase from RRBS protocol (cost-effectiveness, reduced amount of DNA required)
Ryan et al. (2018)	United States of America	Cross sectional	397 women	Peripheral blood	Horvath clock	21.7	increase of epigenetic age acceleration with parity (*p* = 0.005)
Carroll et al. (2021)	United States of America	Prospective longitudinal	33 women	Peripheral blood	Horvath; Levine; GrimAge DNAmPAI1 DNAmTL	33.6	Sleep deprivation at 6 months after delivery causes an increased age acceleration according to Horvath clock (*p* = 0.031) and Levine clock (*p* = 0.001)
Nwanaji-Enwerem et al., 2021a	United States of America	Prospective longitudinal	238 children 483 total observations	Peripheral blood	Horvath; Hannum; SkinBloodClock, PhenoAge, GrimAge	7, 9, 14 ys	Mothers with 1–2 ACEs had children with 0.76-year greater Horvath clock EAA (*p* = 0.004) than those without ACEs
Herrero-Roldán et al. (2021)	Spain	Cross sectional	50 NG-87 CG	Buccal samples	PhenoAge	31.4 vs. 34.7	Higher PhenoAge acceleration age in NG than CG
Kanney et al. (2022)	United States of America	Cross sectional	137 mother-child dyads (GDM vs. non GDM)	Peripheral blood	Hannum, Horvath clock	35.7–6.5 vs. 32.2–7.5	HDL-C significantly associated with EAA in mothers (p 0.006). GDM-mothers epigenetically older than non-GDM group
Shiau et al. (2021)	China	Observational case-control	1,156 mother-child pairs	Peripheral blood of children	Horvath et al. (2013) Hannum clock	30.1 vs. 5.9	According to Horvath and Hannum clock, average DNAm age was higher in the GDM group compared to the non-GDM group (Horvath: 7.23 ± 1.82 vs 6.63 ± 1.87, *p* < 0.0001). Age acceleration was greater in GDM exposed group than the non-GDM-exposed (Horvath: +7.2 months, *p* < 0.0001; Hannum: +16.8 months, *p* < 0.0001)
Nishitani et al. (2022)	Japan	Cross-sectional	51 women	Saliva	Horvath clock Skin&Blood	34.8	They noted that multiparity (less than 4 newborns) encompasses with a process of mAge deceleration (*p* = 0.02) and that precuneus volume increases as mAge deceleration occurred

*p: *p*-value; Ageaccel: age-acceleration; EOA: early ovarian aging; NOA: normal ovarian aging; ys: years; CC: cumulus cells; MGC: mural granulosa cells; DunedinPoAm: Dunedin Pace of Aging methylation.

* GA gestational age; PAA placental age acceleration, EAA epigenetic acceleration, ACE adverse children events; NBW: normal birthweight; ELBW extremely low birth weight; RRBS: representation bisulfite sequencing; NG: neglected group; CG: control group.

## 2 Epigenetic clocks

Epigenome has ushered a new era of molecular research in the field of aging (Erema et al., 2022) as DNA methylation (DNAm) has emerged as one of the most powerful strategies to predict chronological age (Horvath and Raj, 2018; Oblak et al., 2021; Simpson and Chandra, 2021). The precision of this instrument is astonishing, to the point to be used for forensic purposes to estimate chronological age in burned and defaced corpses or in biological specimens (Freire-Aradas et al., 2022). The availability of a precise predictive tool of chronological age can also allow to identify subjects whose chronological and biological age diverge and investigate the health consequences of this variation.

DNA methylation refers to the addition of a methyl group to the 5’ end of DNA under the action of DNA methyltransferase (DNMT), thereby affecting the regulation of gene expression. Usually, this modification occurs on cytosine (C)—phosphate (p)—guanine G) (CpG). A section of DNA rich in CpG in the genome is called a CpG island, with a length of 300–3,000 bp. CpG islands are usually located in the promoter region of the gene, so the CpG island of the gene promoter is a common location where methylation occurs. Approximately 70%–80% of the CpG in the human genome is methylated but its status can vary according to the aging process: CpGs undergo hypermethylation in the promoter regions, while they become hypomethylated in others (Saul and Kosinsky, 2021; Duan et al., 2022). DNAm can be measured by different methods: microarrays (Horvath et al., 2014), pyrosequencing (Zbieć-Piekarska et al., 2015), quantitative PCR and next-generation sequencing methods (Lu et al., 2018). Advances in microarray technology, biostatistics development and the emergence of a new open-access scientific mentality finally allowed the development of epigenetic clocks, the new metrics of biological age based on DNAm patterns (Horvath and Raj, 2018). Epigenetic clocks derive from machine learning models that automatically select the most informative CpGs by regressing (i.e., penalized, cox, elastic net regression model) a transformed version of chronological age on a set of CpGs (Horvath and Raj, 2018; Rutledge et al., 2022). Even if the large number of CpG included may be a guarantee of higher accurateness, a moderate number of CpGs could be robust enough, as epigenetic clocks depict comprehensive properties of the methylome (Duan et al., 2022). This notion has led to the development of simplified and less expensive epigenetic clocks.

**FIGURE 1:**
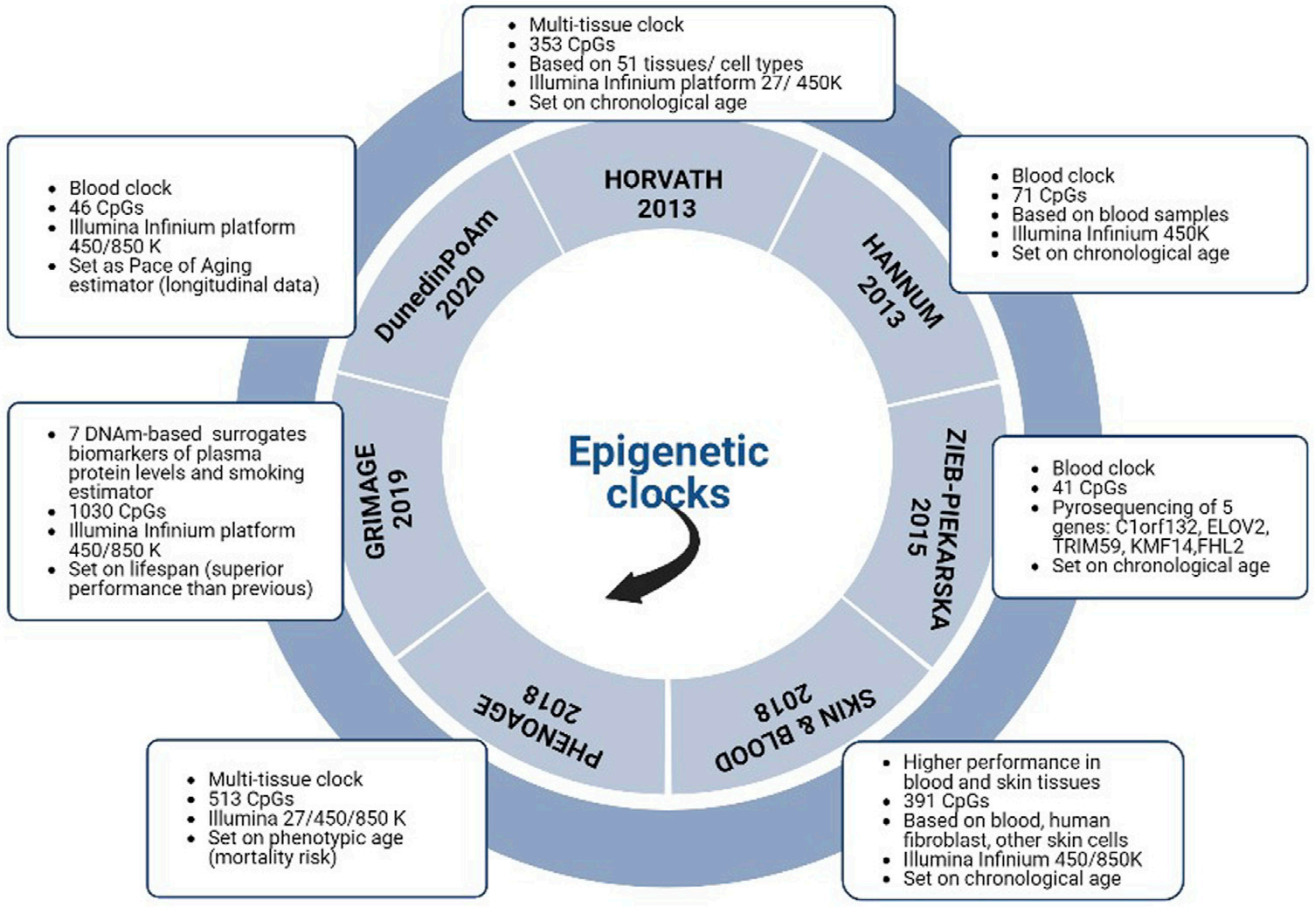
Description of the characteristics of the most common epigenetic clocks.

Epigenetic clocks may be based on one tissue (single-tissue clock) so that their accurateness is permitted only if applied on the tissue type on which they were trained (Hannum et al., 2013) or they could apply to all tissues and cell types across the entire duration of the human lifespan (multi-tissue clock) (Horvath et al., 2014; Levine et al., 2018; Lu et al., 2019). Huge differences in biological age exist among different tissues. The first multi-tissue predictor of age (Horvath Clock) was devised using n = 8,000 samples from 82 Illumina DNA methylation array datasets and encompassing n = 51 healthy tissues and cell types (Horvath et al., 2014). Since 2012, multiple models have spread throughout the world (Oblak et al., 2021; Shahal et al., 2022). While the first-generation clock goal was to be accurate in chronological age prediction (Horvath et al., 2014), the second ones aimed to investigate a possible causative role of DNAm in the ageing process and onset of age-related disorders (i.e., GrimAge, PhenoAge) (Levine et al., 2018; Lu et al., 2019). While GrimAge has been adopted for lifespan prediction, the PhenoAge clock could estimate cancer, or Alzheimer’s disease risk. A multitude of other clocks have spread for different clinical purposes (Levine et al., 2018; Lu et al., 2019; Johnstone et al., 2022). The possible difference between a person’s chronological age and the biological one, provided by epigenetic clocks, is defined as “age acceleration or deceleration”. If predicted DNAm age is greater than chronological age, age acceleration will be positive, if the predicted biological age is less than chronological one, it will be negative (decelerated age). While genetic and inner process could cause “intrinsic” age acceleration (Horvath and Raj, 2018), lifestyle and environmental factors may be detrimental for “extrinsic” one (Franzago et al., 2022). Not only epigenetic clocks may provide a clue on the impact of modifiable factors on the natural process of aging, but also they may give a glimpse of possible effective strategies to make the clock tick backwards (Levine et al., 2018). In fact, the growing awareness of the modulation role of nutrients and other food components on DNAm patterns has paved the way for new studies on the relationship between lifestyle interventions and epigenetic aging (Mathers et al., 2010; Horvath et al., 2014; Zannas et al., 2015; Quach et al., 2017; Franzago et al., 2022). Intriguingly, folates, a standard supplementation both in the pre-conception period and during early pregnancy, have been recently investigated in the aging field (Sae-Lee et al., 2018; Monasso et al., 2021; Nwanaji-Enwerem et al., 2021a). Chanachai Sae-Lest et al. observed that folic acid plus vitamin B12 intake can provide a different effect depending on MTHFR genotype, proving a decreased epigenetic age in women with the more common 677CC genotype (Sae-Lee et al., 2018). Owing to the relevance of the topic and its practical implications, these preliminary data corroborate the need for solid evidence. A description of the main features of most common clocks is provided in Figure 1.

## 3 Infertility

The worldwide trend toward delayed parenthood to the late thirties and even beyond has boosted the focus of reproductive medicine on the possible impact of aging on conceiving success (De Geyter et al., 2020). Women’s natural fertility sharply declines after 35 years and terminates at a median age of 41–42 years (Eijkemans et al., 2014). The subsequent 10 years until the advent of menopause are still characterized by ovulatory cycles, but the quality of the recruited oocytes is incompatible with live births (te Velde and Pearson, 2002). The lost of fertility is not due to a reduction of the remnant ovarian reserve but, conversely, to the deterioration of oocytes quality. Concepts like diminished ovarian reserve and ovarian aging have become “daily bread” for experts dealing with infertility issues. However, clarifying the real meaning of ovarian aging represents a worthwhile endeavor. In fact, despite the increasing number of studies on this topic, the exact biological changes occurring within ovaries overtime are not completely known. The current social trend for couples to delay childbearing has given more urgency to the need of investigating age rhythm and ‘ageing oocyte’ (Ge et al., 2015). Increasing literature has suggested that female reproductive timeline could be somehow punctuated by epigenetic mechanisms and clocks rhythm (Das and Destouni, 2022).

One of the most stimulating theories supports a new concept of age-related infertility as the first sign of an “accelerated aging disease” (Llarena and Hine, 2021; Loose et al., 2022). In other words, ovaries could be the first compartment to show signs of a general process of accelerated aging. The result would be subfertility or even infertility. Thus, infertility could be remarked as part of a more general systemic illness that is defined by accelerated or premature aging process. Proving that infertility could be associated with age acceleration of a person’s biological clock may revolutionize reproductive medicine. Fertility status would no longer concern only couples desirous of offspring, but it could become a matter of global health, also involving the general practitioner who faces a young woman not yet seeking pregnancy. Prompt identification of women at higher risk of earlier exhaustion of fertility could provide them with a double opportunity: on one hand if it is a contemplated wish, they may have time enough for scheduling their family building plan; on the other one, they could prevent the raise of age-related disorders such as cardiovascular or bone health problems. A prompt family building plan could consist of two different chances: anticipating their offspring search time or overcoming the ageing process by freezing eggs or ovarian tissue.

Not at last, biological aging biomarkers could glean additional information to traditional procreative testing (Morin et al., 2018; Monseur et al., 2020; Li Piani et al., 2022). Moreover, as at current state no consensus exists on whether the available traditional ovarian markers could be adopted for a large-scale screening of reproductive senescence, they could be evaluated for this purpose too. Noteworthy, the advantage of the availability of a measurement of ovarian biological age is not limited to a diagnostic purpose. It may also become a precious tool to predict the success of Assisted Reproductive Techniques (ART). Women with predicted compromised oocytes could indeed avoid embarking in the expensive, burdensome, and risky ART journey. They could be straightly advised for egg donation. Apart from these appealing potential clinical applications, studies on the topic are yet initial and insufficient. Available evidence is from here on reviewed, taking into consideration separately the endometrial, peripheral, and ovarian districts.

### 3.1 Results from endometrium

Endometrium is a unique dynamic tissue that cyclically sheds, rejuvenates, and endures complex modifications aimed to promote embryo implantation. However, uterine ageing is often left out of the picture of reproductive female timeline, so that few and contrasting data are available on age-related functional decline in the uterine tissues (Deryabin and Borodkina, 2022). Epigenetic mechanisms are however emerging as key players in regulating cyclic modulation of endometrium (Retis-Resendiz AM et al., 2021). The increase of the global acetylation of some histones (i.e., H3K9ac, H2AK5ac, H3K14ac, and H4K8ac) during the early proliferative phase may be supportive of the regeneration of the endometrial functional layer (Munro et al., 2010), while genes associated with transcription regulation (i.e., ID2, NFAM1, RUNX3, ZNF57) are more methylated in the secretory phase (Saare et al., 2016). In addition, gynecological diseases such as endometriosis may be linked to DNA methylation alterations (Leap et al., 2022). There is evidence of a perturbed biological age of the endometrium in affected women (Guo, 2009; Houshdaran et al., 2016; Saare et al., 2016) and altered speed of epigenetic clocks may predict the progression of some endometrial pathologies, including recurrent implantation failure and endometriosis (Deryabin and Borodkina, 2022).

In 2013, Horvath reported that endometrial epigenetic age badly correlated with chronological one (cor = 0.55), (Horvath et al., 2014). In fact, when the multi-tissue Horvath clock was applied to healthy endometrium samples in the test data, the authors found an high error rate in the chronological age prediction, the endometrium being much younger based on epigenetic data (Horvath et al., 2014). However, Olesen et al. who specifically focused on the endometrium did not confirm this evidence (Olesen et al., 2018). Considering endometrial biopsies sampled at the same point in two consecutive cycles, these authors also described a good correlation between endometrial epigenetic and chronological age, as well as a modest inter-cycle variability. Endometrium was characterized by higher epigenetic acceleration (4.4 years higher than chronological age, *p* < 0.012) and no difference was detected across the different cycles. The authors also postulated that endometrial DNA methylation profile might change according to the phase of menstrual cycle (Olesen et al., 2018). In fact, the bad correlation found in Horvath’s work could derive from the insufficient attention given to the phase of the cycle for the assessment. However, the small sample size of the study of Olesen et al. (n = 9) hampers robust conclusions (Olesen et al., 2018). The cyclic variation of endometrium, its hormone-related nature, and the easy availability of samples all encourage deeper investigations to expand current understanding of the relation between DNA methylation and endometrial aging and function. The uterine factor still represents a neglected topic that should be part in the comprehension of age-related female fertility decline (Deryabin and Borodkina, 2022).

### 3.2 Results from peripheral leukocytes

Most common models have been validated on peripheral blood cells, and in particular in leukocytes. This font of DNAm was used to test the possibility that biological age, more specifically age acceleration, could better explain fertility decline variability than chronological age alone.

In a pilot single-center cohort study, Monseur et al. evaluated the epigenetic age in the peripheral blood cells of 39 women undergoing ART. They used a modified Hovarth clock (they added some intergenic sites) to investigate whether age acceleration could be associated to fertility biomarkers (Monseur et al., 2020). They observed that not only chronological but also biological age negatively correlated with remnant ovarian reserve and oocytes yield. Women with age acceleration showed lower levels of anti-mullerian hormone (AMH) (1.29 vs. 2.29 ng/mL, *p* = 0.05), lower antral follicular count (AFC) (8 vs. 14.5, *p* = 0.05), lower number of oocytes retrieved (5.5 vs. 14.5, *p* = 0.002) and lower number of two pronuclear zygotes (3 vs. 7, *p* = 0.007), compared to the women without age acceleration. However, the design of this pilot study did not allow the authors to compare the predictive capacity of age acceleration with that of common biomarkers of ART success. Moreover, they could not disentangle whether age acceleration may provide independent information on the quality of the oocytes (Monseur et al., 2020). Apparently in contrast, Christensen et al. did not yield any indication of accelerated aging in n = 55 young women with idiopathic early ovarian aging (defined as: minimum two cycles with ≤5 oocytes obtained by ovarian stimulation with at least 250 IU recombinant FSH), when adjusting for chronological age (Christensen et al., 2021). However, the small sample size and the lack of adjustments for other variables (i.e., BMI, smoking) limit the value of the study.

Another possible hypothesis postulates that the information provided by epigenetic clocks may go beyond ovarian reserve. In our latest project, we adopted Zbieć-Piekarska epigenetic model, that is set on five genes and based on pyrosequencing technique (Li Piani et al., 2022). In a group of n = 181 infertile patients aged 37–39 years, we could confirm that women who subsequently conceived and delivered after ART were epigenetically younger than those who did not (respectively: 36.1 ± 4.2 and 37.3 ± 3.3 years, *p* < 0.04). In contrast with Monseur et al. and Lee et al. (Monseur et al., 2020; Lee et al., 2022), we verified that adjusting for the variables that differed between the two groups (AFC, FSH, number of oocytes retrieved) the difference remained statistically significant (*p* = 0.028). Although these findings should be considered with caution for some aspects (restricted patients’ age, choice of the epigenetic model), they suggest that the contribution of epigenetic clocks may not be limited to reflect ovarian reserve. They may capture other aspects of reproductive health (Li Piani et al., 2022).

In 2022, the role of age acceleration was investigated in a large population of pregnant women and using five different epigenetic aging clocks (DunedinPoAm, PhenoAge, DNAmTL, Hannum clock, Horvath Clock) (Lee et al., 2022). The authors considered a much greater sample size, comparing pregnant women conceiving naturally (n = 1,000) with those who conceived after ART treatment (n = 995 patients) (Lee et al., 2022). They noted that ART mothers aged 0.021 years faster than non-ART ones (*p* < 0.03), when the DunedinPoAm clock was adopted. They also found that the etiology of infertility might interplay with epigenetic clock behavior: women with tubal, anovulatory, or idiopathic infertility factor were epigenetically older than the non-ART counterparts (Lee et al., 2022). According to the authors, DunedinPoAm could surpass the other biomarkers in the field of reproductive medicine for its nature of “pace of aging” estimator. It is the only one that reflects aging pace from the time of sample to 10–15 years back and it is more sensitive to weigh environmental exposure burden. This large study stands out from the others as it has included fertile women as control group, it has recruited pregnant women, and it has pioneered novel epigenetic model. It has however some limitations. The authors did not report any information regarding the ovarian reserve, and, by study design, they excluded women with severe infertility that could not be overcome by ART. Finally, data could not be generalized to unpregnant women (Lee et al., 2022).

### 3.3 Results from ovaries

Ovaries could be renamed as “women’s heart”, from a reproductive point of view. They are a time-limited forge of hormones and substances that are crucial for the general wellbeing. Follicular granulosa cells are responsible for the declining of ovarian steroid production associated with reproductive age and they are engaged in an intense crosstalk with the surrounding oocytes. Granulosa cells are crucial for supporting oocytes during follicular growth, maturation, and estrogen production. While epigenetic clocks have been widely validated on easily accessible tissues, their application to several other body regions including the ovaries has been quite limited. Intriguingly, Horvath clock performance seemed lower when tested on hormone-dependent tissues, such as endometrial and breast tissues (Horvath et al., 2014). More specifically, they resulted epigenetically older. Thus, it is not surprising that ovarian microenviroment has been attractive for studying epigenetic clock performance.

#### 3.3.1 Epigenetic aging patterns may be distinct in hormonally responsive tissues

The first study that investigated ovarian epigenetic age in infertile women was published in 2018 (Morin et al., 2018). The authors applied the Horvath model to leukocytes and cumulus cells of women who underwent ART. They divided the enrolled subjects in four groups according to chronological age and ovarian response (A: young good responder; B: young poor responder; C: old good responder; D: old poor responder). They showed that the compartment of cumulus granulosa cells followed a different methylation trajectory with age compared to leukocytes: these cells resulted extremely younger, without difference among the groups for either chronological age or ovarian response. Not at last, they noted that this result was in line with a longer length of telomeres, which represents another valid aging biomarker. Overall, the results suggested a potentially distinct epigenetic timeline in the ovarian compartment. Inferences should however be drawn with caution since the sample size was not large (n = 77) and the authors could not include a control group of fertile women (Morin et al., 2018).

Two years later, the same group sought to verify their results in a larger sample size, recruiting n = 175 women who were undergoing ovarian stimulation and divided into two groups (good responders: >5 oocytes retrieved, poor responders: <5 oocytes retrieved) (Hanson et al., 2020). To note, the definition of ovarian reserve was different compared to their previous work (Morin et al., 2018). The authors confirmed their previous observation: cumulus granulosa cells remained epigenetically younger than the chronological age (average 8.6 ± 2.1 vs. 35.3 ± 4.1 years) when Horvath model was applied. Going further, they attempted to generate an epigenetic model based on cumulus granulosa cell DNA methylation signature, but it turned out to be unsuccessful (Hanson et al., 2020).

All these data also convey the idea that epigenetic aging patterns may be distinct in hormonally responsive tissues. As aforementioned before, Horvath model had a bad performance in case of endometrial tissues, that resulted epigenetically older (Horvath et al., 2014) or it could vary according to the intermenstrual period (Olesen et al., 2018). Cumulus granulosa cells resulted conversely epigenetically extremely younger, and the evidence of a higher telomere length, that is another common aging biomarker, comforted this finding (Morin et al., 2018; Hanson et al., 2020).

#### 3.3.2 Ovaries may work according to a different epigenetic clock

Granulosa cells may reflect a different ageing process than other tissues: they are silent for decades, and then rapidly proliferate from 10–15 quiescent granulosa cells in primordial follicles to 60 million in the ovulatory phase. Given these premises, Olsen et al. postulated that ovarian compartment has a distinct epigenetic heritage than other somatic tissues (Olsen et al., 2018). The authors investigated the process of aging in mural granulosa cells, which are part of the follicular compartment (cumulus granulosa cells being those cells in close contact with the oocytes and mural granulosa cells being the main component of the follicular wall). Data from n = 118 women providing peripheral leukocytes and n = 59 women providing mural granulosa cells were tested using genome-wide methylation analyses (Olsen et al., 2018). Along with Horvath clock and Skin and Blood clock (Horvath et al., 2014; Horvath and Raj, 2018), a new epigenetic model (Granulosa Cell clock), developed by the same authors, was tested on mural granulosa cells (Olsen et al., 2018). As an upgrading of the popular Skin & Blood clock, the Granulosa Cell clock consisted of 296 CpG sites and yielded an improved correlation with chronological age of 0.85 (*p* = 4.3 × 10^−34^) when applied to leukocytes (Olsen et al., 2018). Mural granulosa cells were confirmed to be quite younger (an average of 2.7 years based on Horvath clock) (Olsen et al., 2018). The findings are mainly in line with those emerged from the study by Morin et al. (Morin et al., 2018). These latter authors also reported on cumulus granulosa cells and showed an average of 6.8 years based on Horvath clock and an average of 2.7 years based on Skin and Blood clock (Morin et al., 2018). In both studies, cumulus cells and mural granulosa cells epigenetic age did not reflect chronological age (Morin et al., 2018; Olsen et al., 2018). Besides, in mural granulosa cells, age acceleration difference was negative and the gap with chronological age was larger along with older age. To note, the rate of epimutations in terms of accumulation of DNA methylation errors was higher in advanced maternal age, but its entity was much more considerable in mural granulosa cells than in the corresponding peripheral leukocytes (*p* < 0.003) (Olsen et al., 2018).

#### 3.3.3 Ovarian epigenetic clock may also reflect the traditional concept of ovarian reserve

Olsen et al. applied the Granulosa Cell and Skin & Blood clocks to verify whether epigenetic age could also depict the magnitude of a woman’s ovarian reserve (Olsen et al., 2018). A higher number of epimutations in terms of greater DNA methylation variability characterized mural granulosa cells of women with diminished ovarian reserve. More specifically, variability in epimutations involved AMH and IGF2 genes, both coding for proteins that are involved in folliculogenesis. These epimutations were also higher in advanced maternal age, suggesting that women with diminished ovarian reserve are similar to older women (Olsen et al., 2018).

Symmetrically, Knight et al. proved a significant association between epigenetic age derived from granulosa cells and traditional ovarian reserve markers (Knight et al., 2022). The Grim Age clock was chosen as independent from chronological age, so that it could provide a unique perspective to evaluate oocyte competence. In a group of n = 70 women undergoing ART treatment, the GrimAge acceleration was negatively associated with AMH levels (*p* = 0.003), AFC (*p* = 0.0001), number of mature oocytes retrieved (*p* = 0.0003) and number of fertilized oocytes (*p* = 0.008) (Knight et al., 2022).

These findings, however, deserve confirmation. Indirect findings from studies not designed to address this issue are less trenchant (Li Piani et al., 2022). In addition, one must weigh the clinical relevance.

Overall, increasing literature has focused its attention on the application of epigenetic clocks to the different tissues of female reproductive organs. However, solid knowledge is not available yet and deeper investigations should be encouraged.

## 4 Pregnancy

It is not surprising that the world of pregnancy has been attractive for potential applications of epigenetic hallmarks too (Dieckmann et al., 2021). As a progress of the earlier fetal origin of adult diseases (FOAD) hypothesis (Barker, 2007), the Developmental Origins of Health and Diseases (DOHaD) theory establishes that the interaction at an early stage (from fertilization to early childhood) between genome and external factors (i.e., nutrition, stress, endocrine-disrupting chemicals) may be detrimental for non-communicable disease risk later in life (Arima and Fukuoka, 2020). These theories are based on the ‘developmental plasticity’ assumption, whereby organisms exhibit ‘plasticity’ to master an optimal fit between phenotype and environment, throughout critical developmental periods (Bateson et al., 2004). As this is a time-limited phenomenon, intrauterine “programming” has been the focus of several studies, where epigenetics has emerged as one of the most exciting themes (Bird, 2002; Hochberg et al., 2011). Prenatal and early postnatal periods represent windows of “epigenetic susceptibility” (Coussons-Read, 2013; Reimann et al., 2022).

All these premises underscore the utility of epigenetic clocks to investigate “epigenetic programming” during the early developmental period, as epigenetic clocks are reliable epigenetic footprint. In case of advanced maternal age, epigenetic indices could capture unique signatures that go beyond the known higher rate of adverse newborns outcomes risk. Not only, in case of ART, the possible interference with the extensive epigenetic reprogramming in the early embryo could be disclosed by a change in the epigenetic gestational age acceleration (GAA) (Carlsen et al., 2022). Finally, the search of a solid marker for gestational age (GA) definition has become a research priority, as GA is crucial to forecast fetal maturity. Compared to traditional variables (i.e., last menstrual period (LMP), crown-rump length (CRL), or embryo transfer date (EDT) if conceptions are obtained with ART), epigenetic gestational aging could reveal a higher performance.

Despite the interest that this area deserves, studies are underrepresented and what is lacking is a state of the evidence to date. In the following sections, we will discuss the results gathered according to the tissue included (placenta, cord-blood, peripheral blood) and finally the possible effects of pregnancy on mother’s epigenetic rhythm.

**FIGURE 2:**
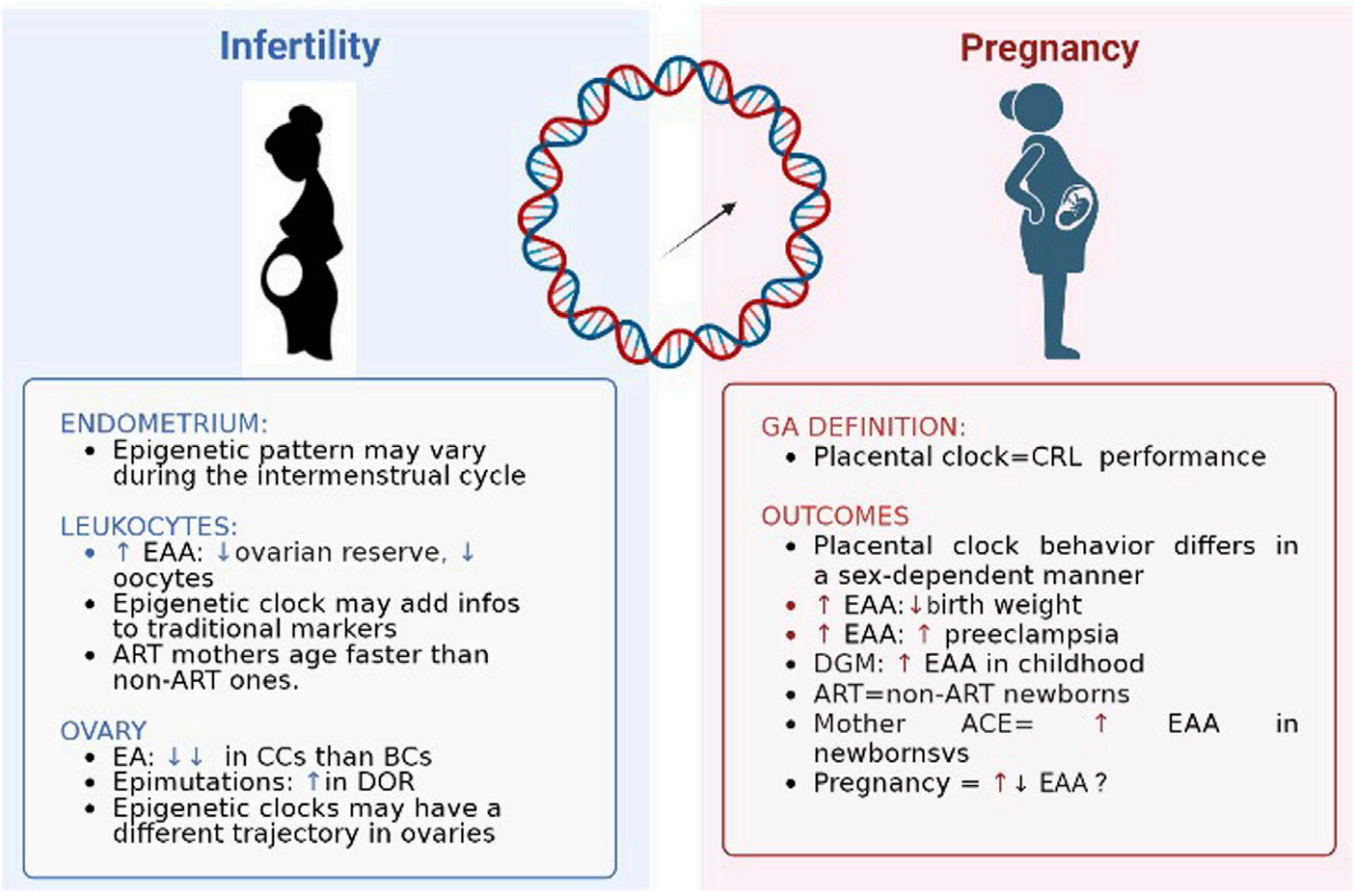
Epigenetic clocks and two mirrors of female fertility time. EAA: epigenetic age acceleration; ART: assisted reproductive technique; EA: epigenetic age; CC: cumulus cells; BC: blood cell; DOR: diminished ovarian reserve; CRL: crown-rump length; DGM: gestational diabetes.

Figure 2 shows a summary of findings regarding epigenetic patterns in case of the two mirrors of fertility time: infertility and pregnancy.

### 4.1 Results from placenta

Placenta is a highly specialized organ that orchestrates the whole course of pregnancy thanks to its multiple functions (i.e., gas and nutrients exchange, immunity transfer, hormone secretion), allowing the normal growth and development of the fetus. Conversely, placental pathology or dysfunction may be the origin of some high-risk pregnancies or fetal adverse events (Gude et al., 2004). It is reasonable that placental DNA methylation profile could provide information about how epigenetic variations ‘work under the skin’ during pregnancy to influence obstetric outcomes (Clark et al., 2022).

Mayne et al. investigated the precise changes in DNA methylation that occur in the placenta across gestation and determined whether DNA methylation data could be used to predict the GA of a placenta (Mayne et al., 2017). Including placental tissue samples from 12 different datasets of only healthy singleton pregnancies (n = 387), the authors developed their placental GA calculator (DNAm GA) based on 62 CpG sites. The performance of DNAm GA sorted out excellent, with high correlation with the chronological age (*p* < 0.001) (Mayne et al., 2017). Starting from the results provided by Mayne et al., Lee et al. used a 6 times larger placental training set (n = 1,102) to create a new more robust placental clock, that could be well suited for GA estimation (Lee et al., 2019). Intriguingly, they also proved that placenta is quite distinct from other tissues regarding DNAm estimators’ application, observing no correlation between their placental GA model and other common epigenetic clocks (Lee et al., 2019). The reliability of these two models was also tested in a following sample of n = 427 placentas, founding an overall satisfactory accurateness and confirming the superiority of the model from Lee et al. (Lee et al., 2019) on the one by Mayne and coworkers (Mayne et al., 2017) (Dieckmann et al., 2021).

Whether on one hand placental epigenetic clocks may be intriguing as a possible marker for GA estimation, on the other they could glean additional information in case of pregnancy-related complications. Indeed, it is feasible to speculate that a state of either maternal or fetal biological disorders may lead to disrupted placental epigenetic ageing, since it is the primary interface and balancing system between the maternal-fetal compartment in pregnancy.

While Lee et al. proved that the DNAm GA performance was not impaired by common adverse conditions, the clock by Mayne et al. under/overestimated GA according to pregnancy conditions (Mayne et al., 2017; Lee et al., 2019). When comparing n = 70 placentas from preeclamptic pregnancies with n = 62 placentas from uncomplicated ones, Mayne et al. showed different methylation pattern in 741 CpG sites. They also underlined that only in case of early-onset preeclampsia, placentas were characterized by accelerated placental aging (*p* < 0.001) (Mayne et al., 2017). Systematic deviations of this clock might reveal interesting biological effects (Mayne et al., 2017). In fact, their result regarding preeclampsia is in line with the most supported theory according to which early preeclampsia may reflect a pathological distressed placenta, while late preeclampsia could be seen as the effect of a maternal status (Hales and Barker, 2001; Valensise et al., 2008; Staff, 2019). This result could be strengthened by an apparent opposite finding provided by Workalemahu et al. (Workalemahu et al., 2021) In n = 312 placentas, the authors found an association between placental epigenetic age deceleration with a lower cardiometabolic profile of the mother (pre-pregnancy BMI, gain of weight, maternal blood pressure). In multivariable-adjusted models, each 1 kg/week increase in their weight was significantly associated with 1.46 (95%CI: −2.61, −0.31), 1.70 (−3.00, −0.40), and 1.71 (−3.11, −0.32) week lower placental epigenetic age acceleration, for each trimester respectively. It could be indeed postulated that the placental immaturity, usually connected to maternal risk factors, may be reflected by an epigenetic deceleration. According to this vision, only early-onset preeclampsia would be characterized by placental age acceleration, and not the late-onset one, that is usually the consequence of maternal adverse profile and would be characterized by the opposite effect, such as age deceleration (Workalemahu et al., 2021).

Moving forward, also sex-specific fetal growth differences and consequent early origins of neonatal and long-term health disparities have been explored also from an epigenetic point of view. Placental acceleration aging behavior differed in a sex-dependent manner (Dieckmann et al., 2021; Workalemahu et al., 2021), proving to be detrimental for fetal weight in males rather than in females (Mayne et al., 2017). Two different explanations could be plausible. First, it could be due to the higher *in-utero* energy demand in male fetuses. Alternatively, female fetuses may display a more efficient risk averse response against the ensuing accelerated aging (Mayne et al., 2017). It is also easily comprehensible that this relation is more manifest at later gestational periods that are typically distinguished by greater fetal oxygen and nutrients demand as well as higher oxidative stress (Tekola-Ayele et al., 2019). Considering n = 408 placentas from the Extremely Low Gestational Age Newborns cohort, Clark et al. revealed differences according to maternal race. In fact, among infants born to black mothers, smoking during pregnancy was associated with higher placental GA acceleration than in other ethnic groups (+0.89 weeks, 95% CI: 0.38, 1.40) and with the duration of admission in neonatal intensive care units (NICU) (−0.08 days/weeks-GA, 95% CI: 0.12, −0.05) (Clark et al., 2022).

All these data advocate the appropriateness of this novel approach to “old issues” like pregnancy disorders (Blair et al., 2013; Leavey et al., 2017). Nevertheless, some limits should be considered in on-going research. Attention should be addressed to the trimester considered. The DNAm GA proposed by Mayne et al. was based on I-II trimester healthy pregnancy, that could conceal later adverse events in III trimester (Mayne et al., 2017) while on the opposite way, the clock by Lee et al. may encompass some biases due to the training set based on III trimester (Lee et al., 2022). Not at last, as studies in twin pregnancies GA acceleration showed that heritability reached only 57%, environmental factors should be taken into account in the data analysis (Tekola-Ayele et al., 2019).

### 4.2 Results from cord blood

Cord blood could be a suitable model for DNA methylation study, as it contains stem cells, and changes in DNA methylation profile of stem cells are known to likely correlate with GA (Bocker et al., 2011).

In 2016, Knight et al. adopted umbilical cord blood samples derived from n = 1,135 newborns to build an accurate neonatal predictor of GA (Knight et al., 2016). In the same year, from the Norwegian Mother and Child Birth Cohort (MoBa) study, Bohlin et al. confirmed the correlation between their prediction model derived by DNA extracted from n = 1,753 cord blood samples and GA based on CRL or LMP (Bohlin et al., 2016). In both studies, DNAm GA correlated more strongly with clinical GA estimates based on CRL than those based exclusively on LMP (Bohlin et al., 2016; Knight et al., 2016). However, this is expected since ultrasound is generally regarded as the most reliable method for GA estimation amongst practitioners. Not only, cord-blood epigenetic clocks proved to be more precise than those provided on placenta samples (Dieckmann et al., 2021). In fact, they observed higher correlation in DNAm GA provided by the cord-blood clocks (r = 0.77, *p* < 0.001) than by placental ones (r = 0.44, *p* < 0.001) (Dieckmann et al., 2021) and a superiority of the clock by Bohlin et al. on that by Knight’s group (Dieckmann et al., 2021; Daredia et al., 2022).

In a prospective setting, Michaeli et al. designed a new robust epigenetic clock, based on n = 41 cord-blood samples, that showed high concordance with GA determined by common parameters (r = 0.77, *p* < 0.001) (Falick Michaeli et al., 2019). The groundbreaking feature of the work was the choice of reduced representation bisulfite sequencing (RRBS) protocol for DNAm: it provides comprehensive genomic CpG coverage, it requires small amount of DNA and it guarantees a more cost-effectiveness than the 450 K array (Falick Michaeli et al., 2019).

Haftorn et al. also developed a new epigenetic clock (EPIC GA) that outperformed both the previous clocks in terms of GA prediction accuracy (Haftorn et al., 2021). In fact, EPIC clock showed high precision in terms of prediction of GA (*R* ^2^ = 0.724) with a median difference (MAD) of 3.24 days. Performance of clocks from Bohlin and Knight’s groups was lower (respectively: *R* ^2^ = 0.610 MAD = 6.69 days; and *R* ^2^ = 0.406 MAD = 4.55) (Bohlin et al., 2016; Knight et al., 2016).

Besides, Haftorn et al. focused on the possible role of conception mode, comparing n = 200 newborns from natural conception with n = 838 ART ones. Although ART has been postulated to impair epigenetic signature, Haftorn et al. found no sharp different accuracy between epigenetic clocks set on CRL or ETD (Haftorn et al., 2021). Indeed, not only ART newborns were comparable to non-ART ones in terms of age acceleration, but a closer inspection in the specific ART procedure (ICSI vs. conventional IVF, fresh transfer vs. frozen transfer) did not show any significant variation (Haftorn et al., 2021).

In n = 814 mother-child pairs, adopting clock by Knight et al. to cord-blood samples, Girchenko et al. found a correlation between newborns’ GA with some adverse maternal or obstetrics variables: maternal age of above 40 years at delivery, preeclampsia or fetal demise in a previous pregnancy, more than two pregnancy risk factors for preeclampsia or intra uterine growth restriction (IUGR). They showed that adverse maternal characteristics influence the newborn developmental stage and fetal organ and tissue maturation, determining variations in the offspring’s DNAm GA at birth (Girchenko et al., 2017). Conversely, the association of maternal age with newborn’s GA was not confirmed by Daredia et al. (Daredia et al., 2022). Indeed, the authors postulated that also negative acceleration (deceleration) of epigenetic gestational age could reflect either prenatal adverse conditions or developmental immaturity (Daredia et al., 2022), as aforementioned before (Workalemahu et al., 2021).

All these findings are in line with the aforementioned DOHaD theory, according to which specific prenatal epigenetic perturbations of the “developmental programming” stage may have consequences during early life and cause accelerated aging (Simpkin et al., 2015; Reimann et al., 2022). Thus, cord blood DNAm GA could be a useful proxy for assessing developmental maturity (Javed et al., 2016). GA acceleration significantly predicted birthweight (*p* = 0.033) in the study of Knight et al. (Knight et al., 2016), consistent with the idea that DNAm GA may reflect general fetal maturity.

Overall, placenta and cord blood seem quite distinct from other tissues regarding the development and application of DNAm-based age estimators. In clinical practice, one could envisage a plethora of applications: its usefulness for GA prediction could be especially foreseen in women who seek prenatal care late in their pregnancy or are unsure of LMP. As a proxy of fetal maturity, placental or cord-blood GA models could allow easier identification of the right treatment in case of preterm birth. Besides, it could be fruitful as a complemental clinical tool to recognize newborns that could benefit from additional monitoring and care.

Nevertheless, cord-blood and placenta samples are not routinary collected before delivery. This aspect is not a matter of minority, as it would make the device currently unsuitable from a clinical point of view except after birth or confined to research purposes only. However, the presence of fetal cells in the maternal circulation is currently ascertained and may open the opportunity for new models based on cell-free fetal DNA (cffDNA), making GA estimate potentially extremely accurate simply by collecting maternal blood samples at any time during pregnancy. Future studies should also focus on amniocytes obtained with amniocentesis because this is a more accessible and safer modality to obtain fetal cells.

### 4.3 Results from maternal peripheral blood

The bond between pregnancy and DNA methylation profile has been deeply investigated in mother and newborns’ peripheral blood. As fetal experience might permanently influence adult health through “developmental plasticity” (Hales and Baker, 2001; Wadhwa et al., 2009), it is not surprising that fetal birthweight has deserved a great attention. While at first it has been seen as an index of maternal nutrition, later, birthweight has gained the role of general intrauterine health proxy, due to the awareness that multiple factors can influence it (Rondó et al., 2003; Grote et al., 2010). Ross et al. were the first to describe an inverse association between second trimester maternal GrimAge acceleration and gestational length or birthweight (Ross et al., 2020). As part of a longitudinal study designed to test the impact of antenatal maternal mood on pregnancy and *postpartum* outcome (Healthy Babies Before Birth HB3), Ross et al. described the association between GrimAge profile of n = 77 women at their II trimester and newborns’ birthweight and length. The significant correlation between an increase of mothers’ GrimAge acceleration and lower newborns’ birthweight (*p* = 0.001) highlights the potential role of maternal biological aging on birth outcomes. The authors themselves however underline the possible selection bias due to the recruitment of low-risk pregnancies as well as the lack of validation of epigenetic indices in pregnancy (Ross et al., 2020).

Madden et al. corroborated their results, presenting a significant relation between low birthweight and GrimAge acceleration (Madden et al., 2021). Based on a Scottish family-based cohort, the authors conducted an epigenome-wide study of birthweight using whole blood DNA derived from n = 1,757 adult samples. Among the five adopted epigenetic models (Horvath clock, Hannum, PhenoAge, GrimAge, DNAmTL), only GrimAge and DNAmTL revealed a significant increase of epigenetic age along with low birthweight (Madden et al., 2021). The authors postulated that while DNAmTL could reflect aging phenomenon at a cellular level, GrimAge could describe this aspect at a whole-body level. Finally, they could state that GrimAge outperforms the other models in the framework of pregnancy (Madden et al., 2021). To note, results by Madden et al. regarding DNAmTL are in contrast with previous works that excluded a role of telomere system in birth outcome (Akkad et al., 2006) and with data from Ross et al., where DNAmTL seemed independent from birthweight (Ross et al., 2020).

Symmetrically, extremely low birth weight (ELBW≤1,000 g) could provide a model from nature for how early adversity may shape adult phenotypes (Saigal et al., 2020). Adopting Horvath clock to n = 45 ELBW and n = 47 normal birthweight (NBW) adult survivors, Mathewson et al. observed that ELBW survivors had 2.16 epigenetic years more than their NBW peers for each additional adult risk factor (i.e., BMI, respiratory sinus arrhythmia, blood pressure) (Mathewson et al., 2021). The association between epigenetic age and cumulative risks was characterized by an increasing degree over time, reaching higher significance at their 40s (Mathewson et al., 2021). Their numbers supported the idea that extreme perinatal adversity, such as ELBW, may cause perturbations in the “epigenetic maintaining system” and influencing the “developmental plasticity” in a negative way. However, further considerations are limited for some reasons: the reduced sample size, the single time-point nature of the study that does not allow assessment of dynamic changes in epigenetic aging, the missed choice of second-generation models that may be more appropriate for the purpose (Mathewson et al., 2021).

In other projects, epigenetic rhythm disturbances have been exploited as a potential mechanism linking maternal gestational diabetes (GDM) with offspring’s metabolic risk in later life. In a study set on mother-child pairs (n = 1,156), Shiau et al. adopted Horvath and Hannum clock to verify whether offspring exposed to GDM exhibited accelerated epigenetic ageing in their early childhood, compared to those not exposed (Shiau et al., 2021). Even after adjustment for potential confounders, offspring age acceleration remained higher in the GDM group than in the non-GDM group (according to Horvath clock: +4.96 months higher, *p* = 0.0002; Hannum clock: +11.2 months, *p* < 0.0001) (Shiau et al., 2021). Similarly, even if in a smaller sample, Kanney et al. examined the association of epigenetic acceleration with five metabolic biomarkers of interest and GDM (Kanney et al., 2022). In a total of n = 137 mother-child dyads in the Health After Pregnancy - Intergenerational Transmission of Obesity (HAPi) study, the authors compared the result provided by Hannum and Horvath clock in mothers with a prior GDM pregnancy and those without. They could show not only a higher mean epigenetic age in GDM group than in the non-GDM one, but also that among the biomarkers evaluated (leptin, HOMA IR, HDL C, fasting glucose), a statistically significant association was registered between HDL-C and epigenetic age acceleration residuals for the Hannum clock (*p* = 0.0063) in GDM mothers (Kanney et al., 2022). Both these studies showed the effect of a suboptimal prenatal developmental milieu of the offspring and go along with other data that supported a possible impact of hyperglycemia on developmentally crucial genes (i.e., energy metabolism, metabolic regulation), causing persistent epigenetic changes (Bouchard et al., 2012; Reichetzeder et al., 2016). Nevertheless, additional investigation across the life course using longitudinal samples is warranted (Shiau et al., 2021).

### 4.4 Transgenerational effects

**FIGURE 3:**
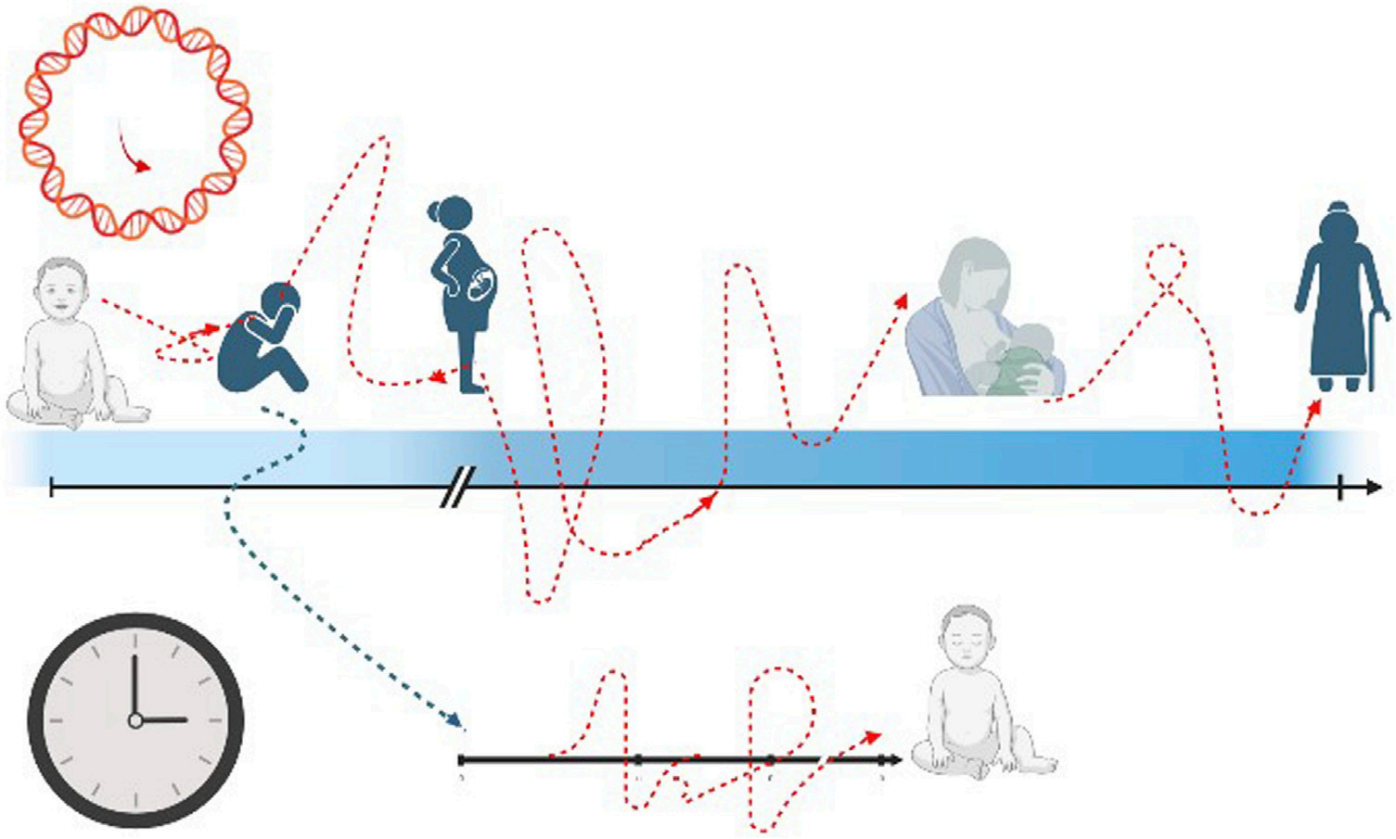
The transgenerational effect of epigenetic pattern. The black arrow and black clock represent the chronological time rhythm, that is linear and straight. The red arrow and red clock stand for epigenetic age with a non-linear way of proceeding, with acceleration and decelerated slopes. The blue arrow figures the possible impact of mother’s past adverse events (even in childhood) on the future newborn’s health.

Moving further and going beyond DOHaD theory, the starting point of ageing process could be placed even before intrauterine lifetime and it could be postulated that the “epigenetic programming and plasticity” could be shaped by maternal experience, even before becoming mother. Adverse child events (ACE) refer to negative psychosocial experiences that individuals experience during the first 18 years of life, that can span from abuse, household dysfunction and neglect (Felitti et al., 1998). There is consistent evidence that ACE could cause important consequences in later health life and a consequent hypothesis formulates a possible intergenerational effect, in part due to epigenetic inheritance (Deschênes et al., 2021). In other words, it is possible that events in the mother’s childhood could have effect during pregnancy and more specifically on her future newborns’ outcomes. Nwanaji-Enwerem et al. verified whether preconception maternal ACE could cause stress-related physiologic reprogramming that might result in child health changes, causing intrinsic epigenetic acceleration age, due to intergenerational effects (Nwanaji-Enwerem et al., 2021b) (Figure 3). Describing ACEs experienced by mothers during their childhood and epigenetic aging of their children at 7, 9, and 14 years, Nwanaji-Enwerem et al. showed that children exposed to adverse events were 0.76-year older than children of mothers who did not report these events, in terms of Horvath epigenetic age acceleration (*p* = 0.004) (Nwanaji-Enwerem et al., 2021a). These findings are the first to exploit the relation between early mother life and epigenetic consequences on their children, and they can provide a molecular basis for the perpetuation of health disparities over time in marginalized populations (Nwanaji-Enwerem et al., 2021b). These data agree and indeed go beyond the results provided by George et al., who showed an influence of social disadvantage exposure in childhood on the biological ageing during adulthood (George et al., 2021). In a cohort of 53-year-old n = 1,376 adults, George et al. exhibited that disadvantaged childhood and lower adult socioeconomic positions were characterized by an accelerated aging process according to GrimAge estimator (*p* < 0.001) (George et al., 2021).

However, another explanation to the data published by Nwanaji-Enwerem et al. may be provided by Herrero-Roldán et al. (Herrero-Roldán et al., 2021). Comparing n = 87 mothers with n = 50 women characterized by “neglect attitude” towards their newborns (neglect group NG), the authors observed that the NG was typically composed by women who have had a disadvantaged background: a higher number of pregnancies in particular at younger age, lower education level, greater childhood maltreatment, propensity for psychiatric disorder, and more economic difficulties. Besides, the authors observed that NG was 2 years epigenetically older than the control group, applying PhenoAge estimator to the collected buccal samples (Herrero-Roldán et al., 2021).

Thus, data from Nwanaji-Enwerem et al. could be justified by two different hypotheses. On one hand, as women with ACE are characterized by epigenetic accelerated age (Herrero-Roldán et al., 2021), their newborns could inherit this disadvantaged epigenetic profile. On the other one, it is also likely that women with ACE experiences are at higher risk of adopting neglect attitude towards their newborns (Herrero-Roldán et al., 2021), causing a prenatal or neonatal adverse environment that would be detrimental for the newborns’ developmental maturity and the cause of their epigenetic acceleration profile.

To sum up, epigenetic clocks further underscore the urgence of providing timely and effective mental health support to vulnerable populations, especially during pregnancy. These last papers illustrate how the mother’s mental health state, that has been mostly “neglected”, can influence the quality of the prenatal environment and consequent multiple aspects of child development (McGill et al., 2022).

### 4.5 Epigenetic effect of pregnancy on mother

As epigenetic clocks have been exploited to detect the impact of pregnancy and maternal characteristic on obstetric and newborns’ outcomes, the possibility that pregnancy may have consequences on maternal health status has raised the interest in epigenetic age behavior, too. In a very preliminary work (n = 33 women), Carroll et al. investigated the effect of sleep deprivation in the *postpartum* phase and on the biological clock (Carroll et al., 2021). Applying Horvath clock and PhenoAge estimator to peripheral blood samples, they observed that insufficient sleep duration during the early *postpartum* period was associated with accelerated biological aging (*p* = 0.03 and *p* = 0.001 with Horvath and PhenoAge) (Carroll et al., 2021). It is not hard to believe a possible trade-off between reproductive effort and life expectancy (Bolund et al., 2016). Reproductive effort could change brain structures involved in the sociability required for parenting, likely influencing both mAge acceleration and lifespan (Nishitani et al., 2022). Based on a cohort of n = 51 mothers aged 27–46 years, Nishitani et al. explored the relation between epigenetic age and the regional gray matter volumes measured by magnetic resonance imaging (MRI). They noted that multiparity (less than four newborns) encompasses a process of mAge deceleration (*p* = 0.02) and that precuneus volume increases as mAge deceleration occurred. However, their findings should be considered in the context of some limitations. Saliva samples did not let the adoption of second-generation epigenetic clock, and nulliparous women should be included in future studies to confirm their results (Nishitani et al., 2022). On the other hand, this study opens new fascinating scenarios. This proof could represent the molecular description of the dynamic structural and functional phenomena during pregnancy, linked to “maternalization” of the brain (Hoekzema et al., 2017; Luders et al., 2020) and could give the clarification of longer life expectancy in child-rearing mothers (less than four children) according to demographic studies.

Evidence is however not univocal. In a cohort of n = 397 20–22-year-old women, Ryan et al. also described a raise of DNAmAge acceleration with parity (*p* = 0.005), consistent with the idea that reproduction comes at a cost of ‘maintenance’ and that epigenetic biomarkers could reflect this “stress” (Ryan et al., 2018). However, they failed to detect an increase of DNAmAge in case of low socio-economic status, a robust “stressor”, even if they justified it as a plausible effect of young age compensation. The narrow age range chosen could indeed introduce some biases (Ryan et al., 2018) that may explain at least in part the difference with previous works (Nishitani et al., 2022).

Gestational epigenetic age clocks are still in their nascent stages. Given that pregnancy emphasizes unique and dynamic physiological adaptations across physiological systems, epigenetic clocks should be validated in pregnant samples. Besides, even if the set cohort homogeneity provides robustness to epigenetic indices, further validation is mandatory in larger and more heterogenous samples to guarantee its generalizability.

## 5 Conclusion and future directions

In this review, we mainly discussed the possible role of epigenetic clocks in the female reproductive health, starting with physiological fertility issues but then inevitably encompassing the linked topics of infertility and pregnancy. The role and mechanisms of aging in female reproductive lifespan has just recently gain scientific attention but multiple aspects are still neglected.

**FIGURE 4:**
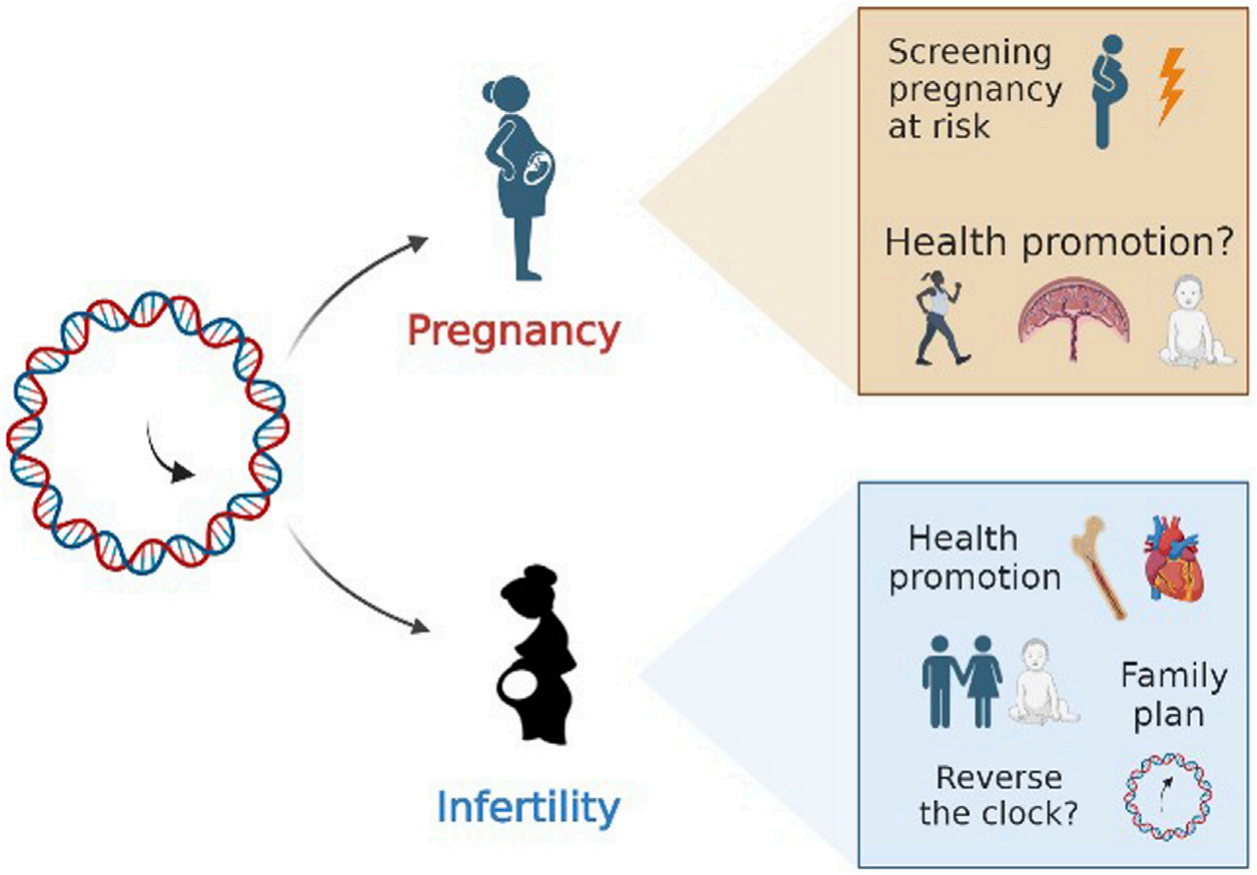
Future directions.

Epigenetic clocks represent a promising research tool for the coming years and may bring us closer than ever to understanding the complexities of reproductive function. It is reasonable that exploiting epigenetic mechanisms may help advance inquiry into the relation between fertility time and aging. In case of infertility, clinical practice could take advantage of epigenetic biomarkers. Biological age could become a precious information in the diagnostic fertility work-up. Women affected by an “accelerated aging” process could be promptly addressed to adequate health promotion programs and provided with the awareness of “family plan” urgency, if it is a desired wish (Figure 4). Similarly, oocyte or ovarian tissue freezing may not only relegated as “social freezing”, but this choice could be part of health promotion and prevention medicine programs, even in those countries where elective egg freezing is limited to small subgroups of women at high risk of ovarian reserve exhaustion such as those scheduled for radio or chemotherapy.

Regarding pregnancy, epigenetic clocks have the merit of opening a new vision to old arguments. Pregnancy may be not confined to a restricted time window for a woman’s life, but may deserve higher consideration for the whole life. The possibility that environmental exposure could deeply impact on epigenetic footprints and determine the epigenetic rhythm of ageing could renovate the way of dealing with pregnancy issues too. Epigenetic clocks may serve as screening tool for high-risk pregnancies, and they may foster improvement of pregnancy outcomes and *postpartum* wellbeing. Not only, but it could represent a new tool for dealing with social disparities and help the support of “vulnerable” pregnant populations. Then the future development of GA estimator based on fetal free DNA in maternal blood could completely revolutionize even the prenatal medicine. Whether these epigenetic patterns are stable or shift over the course of reproductive female life represents another key point in the knowledge that may unveil pragmatic therapeutic approach to “turn back the clock” in (a very) distant future. Last, but not least, there is the need to explore more in depth the possible impact of a history of infertility and ART on the epigenetics of newborns.

Future research should explore the unique epigenetic mechanisms underlying aging, that may play a role in the infertility and the obstetric world, with all the possible consequent implications. Finally, it is important to emphasize that pregnancy and infertility may not be seen as two different “worlds” but could represent “chapters” of a same story, whose red thread is the epigenetic rhythm.
